# Simultaneously Improve Transferability and Discriminability for Adversarial Domain Adaptation

**DOI:** 10.3390/e24010044

**Published:** 2021-12-27

**Authors:** Ting Xiao, Cangning Fan, Peng Liu, Hongwei Liu

**Affiliations:** School of Computer Science, Harbin Institute of Technology, Harbin 150001, China; fancangning@gmail.com (C.F.); pengliu@hit.edu.cn (P.L.); liuhw@hit.edu.cn (H.L.)

**Keywords:** deep leaning, transfer learning, adversarial domain adaptation, image classification

## Abstract

Although adversarial domain adaptation enhances feature transferability, the feature discriminability will be degraded in the process of adversarial learning. Moreover, most domain adaptation methods only focus on distribution matching in the feature space; however, shifts in the joint distributions of input features and output labels linger in the network, and thus, the transferability is not fully exploited. In this paper, we propose a matrix rank embedding (MRE) method to enhance feature discriminability and transferability simultaneously. MRE restores a low-rank structure for data in the same class and enforces a maximum separation structure for data in different classes. In this manner, the variations within the subspace are reduced, and the separation between the subspaces is increased, resulting in improved discriminability. In addition to statistically aligning the class-conditional distribution in the feature space, MRE forces the data of the same class in different domains to exhibit an approximate low-rank structure, thereby aligning the class-conditional distribution in the label space, resulting in improved transferability. MRE is computationally efficient and can be used as a plug-and-play term for other adversarial domain adaptation networks. Comprehensive experiments demonstrate that MRE can advance state-of-the-art domain adaptation methods.

## 1. Introduction

Extensive researches on deep learning has resulted in excellent supervised learning performance for computer vision tasks. However, the prerequisite for the widespread application of deep learning is a great amount of annotated data, which may be hard to obtain due to a large amount of manual labor involved. The direct application of a deep neural network (DNN) that has been pre-trained on existing datasets cannot provide effective generalization in a new domain because of the domain shift problem. To alleviate such labeling efforts and domain shifts, researchers have been resorting to unsupervised domain adaptation (DA) [1,2], which aims to learn a discriminative classifier using source domain data with smaller risks on target domain data in the presence of domain shifts.

Theoretical analysis on DA [3] suggested that the target risk can be bounded by minimizing the source risk and a certain specific statistical discrepancy between the two domains, which has inspired a series of shallow [4] and deep learning-based DA methods [5,6]. Early shallow DA methods strove to learn domain-invariant feature representations or to reweigh the importance of source instances based on their relevance to the target domain [7,8]. Recent deep DA methods have harnessed the power of DNN to extract additional transferable features [9,10]. Such methods commonly minimize a measure of distribution discrepancy [11,12] between the source and target domains. Inspired by generative adversarial networks [13], adversarial DA methods encourage the feature extractor to learn domain-invariant representations by playing the min-max game in an adversarial learning paradigm.

**Figure 1:**
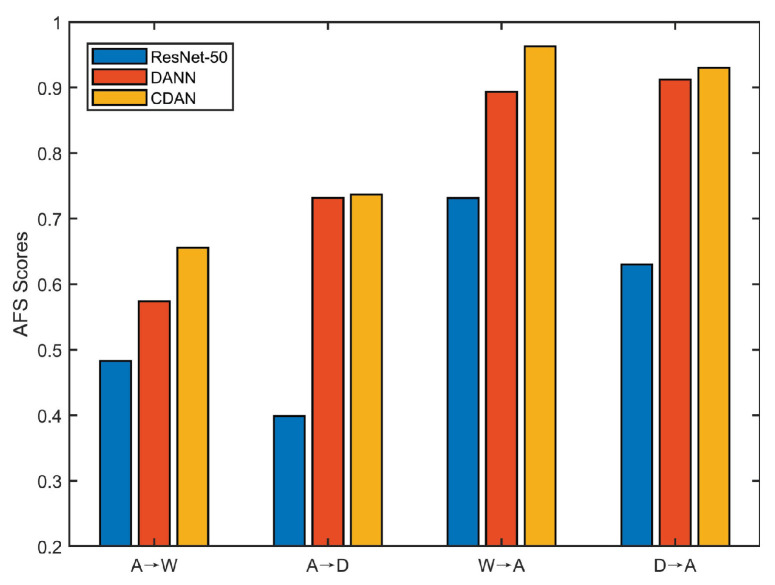
AFS values of different methods on Office-31 dataset, where the horizontal axis are DA sub-tasks and the vertical axis represents the AFS of target feature on its corresponding subtask. In Office-31, domain Amazon, DSLR, and Webcam are abbreviated as A, D, and W, respectively.

Although adversarial DA methods have yielded remarkable improvements, they still exhibit two intrinsic limitations. First, the feature discriminability is inevitably suppressed during the process of adversarial learning of domain-invariant representations, as revealed in [14]. We investigated the discriminability of target domain features extracted from ResNet-50 [15], DANN [16], and CDAN [17] in Office-31 [18] dataset. We applied the angular Fisher score (AFS) [19] to measure the feature discriminability on the target domain, which was defined as:

(1) $$AFS=S_{w}/S_{b},$$

where $S_{w}=\sum_{i}\sum_{x_{j}\in X_{i}}\left(1-\cos\langle m_{i},x_{j}\rangle \right)$ is the intra-class scatter, $S_{b}=\sum_{i}n_{i}\left(1-\cos\langle m,m_{i}\rangle \right)$ is the inter-class scatter. Moreover, $X_{i}$ represents samples from *i*-th class, $x_{j}$ is the feature of *j*-th sample in $X_{i}$, $m_{i}$ is the mean vector over class *i*, *m* is the mean vector over the entire dataset, and $n_{i}$ represents the sample number in class *i*. In general, a lower Fisher value indicates that the features are more discriminative. The preliminary empirical investigation of this limitation is depicted in Figure 1. As we know, the transferability of ResNet-50, DANN, and CDAN is sequentially enhanced. For each subtask in Figure 1, from ResNet-50 to CDAN the AFS value gradually increases, indicating that the feature discriminability sequentially decreases. This implies that the transferability is enhanced at the expense of degraded discriminability in adversarial DA.

Second, the existing adversarial DA methods have not fully exploited the transferability, and they only focus on the distribution matching in the feature space. The data discrepancy in the joint distribution of input features and output labels still lingers in the network. In these methods, either a single domain discriminator is learned to align the marginal P(X) distributions [16,20] or multiple discriminators together with the target domain pseudo-labels [17] are used to align conditional distributions P(X∣Y) between the two domains. Regardless of the marginal distribution or conditional distribution alignment, these methods only focus on domain shifts in the feature space, with little emphasis on domain shifts in the label space.

**Figure 2:**
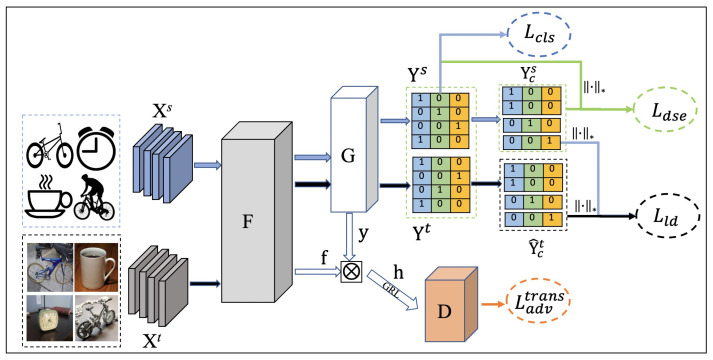
The schematic of our MRE network. $X^{s}$ and $X^{t}$ and are a mini-batch of source and target samples. Suppose there are three classes and the batch size is 4. After samples pass through the feature extractor *F* and the classifier *G*, we will obtain two 4×3 prediction matrices $Y^{s}$ and $Y^{t}$. In $Y^{s}$ and $Y^{t}$ matrix, blue represents class 1 (bike), the green represents class 2 (clock), and yellow represents class 3 (mug). The value in the table indicates the probability (we use the ground-truth prediction for pedagogical reasons) that the sample belongs to the category. ${∥\cdot ∥}_{*}$ represents nuclear norm operator (Best viewed in color).

In this paper, we propose a matrix rank embedding (MRE) method towards transferable and discriminative adversarial DA. Figure 2 presents a schematic illustration of the MRE method. The motivation of MRE is based on the basic philosophy of matrix rank. The rank of a matrix is the maximum number of its linearly independent vectors. High-dimensional data such as images often have a small intrinsic dimension. Thus, multiple class data often lie in a union of low-dimensional subspaces. Data from the same low-dimensional subspace is highly correlated, exhibiting a low-rank structure [21,22,23], while data from different low-dimensional subspaces are not correlated, which exhibits a high-rank structure.

Based on the above observations, given two different domains, we explicitly constrain data from the same class to have the same low-rank structure while imposing a high-rank structure for data from different categories. The optimization of rank-based objectives is NP-hard since it is non-convex. In practice, we use the nuclear norm for a surrogate objective as it is the tightest convex envelope of matrix rank within the unit ball. Specifically, our method is manipulated in the space of probability predictions. Unlike LDA Fisher discrimination measures, by minimizing the nuclear-norm of data in the same class directly and maximizing the nuclear-norm of all data through its connection to the Frobenius-norm, our method reduces the intra-class variation and increases the inter-class separation, resulting in improved discriminability. In addition to aligning the class-conditional feature distributions $P(X|Y_{c})$ (where *c* is a class) across two domains statistically, MRE forces the data of the same class in different domains to exhibit an approximate low-rank structure, thereby aligning the class-conditional label prediction distribution $P(Y_{c})$, resulting in improved transferability. MRE is computationally efficient and can be used as a plug-and-play term for other adversarial DA networks. The empirical results and ablation studies demonstrate that MRE can simultaneously improve transferability and discriminability, resulting in significant performance advancement for adversarial DA.

## 2. Related Work

This paper will focus on deep learning-based DA methods, which can be roughly categorized as discrepancy-based methods and adversarial learning-based methods.

### 2.1. Discrepancy-Based DA

The discrepancy-based method aims to align certain distribution discrepancies between domains in one or more feature layers [24]. These kinds of distribution discrepancies can be maximum mean discrepancy (MMD) [9,10,11,25], central moment discrepancy [26], second-order statistics matching [12], *f*-divergences [27], or the discrepancy of gradients [28]. In general, MMD measures the source and target distributions as the distance between the corresponding mean elements in a reproducing kernel Hilbert space (RKHS). For example, the deep domain confusion (DDC) method [11] applies the MMD loss on the last feature layer and trains the network together with the classification loss. Then, deep adaptation networks (DAN) [9] apply MMD loss on multiple feature layers and minimizes the distribution discrepancy with multiple kernel variants of MMD.

Unlike the above method that eliminates domain distribution discrepancy by aligning the marginal distributions, the joint adaptation network (JAN) [10] proposes to align the joint distributions discrepancy of multiple domain-specific feature layers. Based on MMD, JAN also proposes a new distribution distance criterion, named joint maximum mean discrepancy (JMMD). Then, Sun et al. propose a very simple but effective method CORAL [12] to align the cross-domain distributions by matching the mean and variance between features. In the subsequent research, the maximum density divergence (MDD) [25] proposes to minimize the divergence between domains and maximize the density within the class to align the distribution divergence. In addition to the method of explicitly reducing the cross-domain distribution difference, there is also a method [29] that implicitly minimizes the domain difference by aligning the batch normalization (BN) statistics. Instead of directly manipulating the source and target domain features, the authors of [28] minimize the gradient difference for the two domains.

### 2.2. Adversarial Learning-Based DA

Adversarial learning-based methods minimize the cross-domain distribution discrepancy by playing an adversarial game [20,30,31,32]. The pioneered adversarial DA method, domain-adversarial neural network (DANN) [16], proposes a gradient reversal layer (GRL) to achieve adversarial domain training with standard back-propagation and stochastic gradient descent. Following that, the adversarial discriminative domain adaptation (ADDA) [20] applies two independent mappings for the two domains without sharing weights to achieve adversarial training. With the help of an additional domain classification layer, Tzeng et al. [33] propose a new domain confusion loss, which aims to encourage the classification prediction close to a uniform distribution over binary labels.

In addition to using the discriminator for explicit adversarial training, some papers have avoided using domain discriminators. By estimating the empirical Wasserstein distance of the two domains, Shen et al. [34] propose to minimize the distance in an adversarial way to optimize the feature extractor network. The maximum classifier discrepancy (MCD) [30] method does not explicitly use a discriminator but applies two classifiers to maximize the domain difference to detect target samples outside the support of the source, generating target features near the support to train a feature extractor and minimize the domain difference.

Recent research suggested that feature discriminability plays a crucial role in adversarial DA [35,36], and transferability is enhanced at the expense of deteriorated discriminability [14]. MADA [35] and CDAN [17] integrate the classifier prediction information into adversarial domain training and pursue multimodal distribution alignment. Transferable adversarial training (TAT) [37] enhances feature discriminability to guarantee adaptability. Batch spectral penalization (BSP) [14] preserves discriminability by penalizing the largest singular value of batch features. Domain-symmetric networks (SymNets) [38] construct an additional classifier that is shared by the source and target classifiers for discriminative DA. BNM [39] utilizes F-norm and rank maximization to improve the discriminability and diversity of predictions. The authors of [40] reduce the distribution shifts between classes in different domains from the perspective of class-conditional distribution alignment. These methods only enhance feature discriminability from the statistical perspective and focus on matching the distribution in the feature space, with less emphasis on the label space. In contrast, our method enhances feature discriminability from a geometric perspective and improves transferability by simultaneously aligning the feature distribution in the feature and label spaces.

## 3. Methods

The proposed method mainly consists of two parts. The first is to learn discriminated subspace embedding to improve the feature discriminability, and the second is to align the class-conditional distribution in both feature and label space to improve the transferability. We begin with several notations and the baseline for adversarial DA.

### 3.1. Preliminaries

In unsupervised DA, there is a source domain, denoted as $D_{s}={\left\{\left(x_{i}^{s},y_{i}^{s}\right)\right\}}_{i=1}^{s}$, which has $n_{s}$ labeled samples; and a target domain, denoted as $D_{t}={\left\{x_{i}^{t}\right\}}_{i=1}^{t}$, which has $n_{t}$ samples without annotations. The source and target domains cover *C* classes, where $y_{i}^{s}\in \left\{1,...C\right\}$. The two domains are sampled from their respective joint distributions, with $P_{s}\left(x^{s},y^{s}\right)\neq P_{t}\left(x^{t},y^{t}\right)$. In general, mini-batch training is used in deep learning. Given a mini-batch of source data $X^{s}$ and target data $X^{t}$, we denote the batch size as *N*.

We follow the standard adversarial DA framework, which has a feature extractor f=F(x), a category classifier y=G(f), and a domain discriminator d=D(f). In standard adversarial DA, *D* is trained to distinguish which domain the features come from and *F* is trained to extract domain-invariant features to confuse *D*. The most widely accepted framework for adversarial DA is minimizing the classification error on the source domain labeled data and an additional transfer loss between the two domains. The classification loss in the source domain is formulated as:

(2) $$L_{cls}=-\frac{1}{n_{s}}\sum_{i=1}^{n_{s}}L_{ce}\left(G\left(F\left(x_{i}^{s}\right)\right),y_{i}^{s}\right),$$

where $L_{ce}$ is the cross-entropy loss. The transfer loss can be formulated as:

(3) $$L_{adv}=-\frac{1}{n_{s}}\sum_{i=1}^{n_{s}}\log\left(D\left(f_{i}^{s}\right)\right)-\frac{1}{n_{t}}\sum_{j=1}^{n_{t}}\log\left(1-D\left(f_{j}^{t}\right)\right).$$

Formally, the adversarial DA is formulated as:

(4) $$\begin{array}{cc} \; & \min_{F,G}L_{cls}+L_{adv}\\ \; & \max_{D}L_{adv}. \end{array}$$

### 3.2. Learning Discriminated Subspace Embedding

Cross-entropy loss, together with softmax, is arguably one of the most commonly used classification components in convolutional neural networks. Its decision boundary is determined by the angle between the feature vector and the vectors corresponding to each class in the linear classifier. However, despite its popularity and excellent performance, this component does not explicitly encourage the similarity within classes, nor the separation between classes of the learned features. Moreover, the investigation outlined in the first section demonstrated that the feature discriminability is degraded in adversarial DA methods. Therefore, following the concept of the angle between the feature and classifier vector, a natural strategy for explicitly enhancing the discriminability involves causing the features from the same class to fall into the linear subspace that is well-aligned with its classification vector, and the subspaces corresponding to different features should be separated as far as possible.

In manifold learning, high-dimensional data usually has a small intrinsic dimension, which can be effectively approximated by a low-dimensional subspace of the high-dimensional ambient space [41]. Furthermore, the low-dimensional subspace points to the matrix rank. On this basis, we propose exploiting the matrix rank embedding as the key learning criterion to force samples from the same class to fall into the same subspace, while the subspaces of the data of different categories are separated as far as possible to enhance the feature discriminability.

For the given mini-batch training data $X^{s}$ and $X^{t}$, $Y^{s}=G\left(F\left(X^{s}\right)\right)\in R^{N*C}$ and $Y^{t}=G\left(F\left(X^{t}\right)\right)\in R^{N*C}$ are their prediction matrix by the classifier. Let $Y_{c}^{s}$ be the sub-matrix of the source prediction that belongs to class *c*, and $Y=[Y^{s};Y^{t}]$ is the prediction matrix for the entire mini-batch. To enhance the discriminability, we enforce a low-rank constraint on the data from the same class and a high-rank constraint on the data from all classes, which can be formulated as a discriminative subspace embedding loss $L_{dse}$:

(5) $$L_{dse}=\sum_{c=1}^{C}rank\left(Y_{c}^{s}\right)-rank\left(Y\right).$$

Intuitively, minimizing the first term encourages samples from the same subspace to have consistent predictions, and minimizing the second term (i.e., −rank(Y)) encourages samples from different subspaces to have diverse predictions. The rank function is presented here for pedagogical reasons. We will later replace it with the nuclear norm and show how the nuclear norm increases separations between the different classes. A tensor’s nuclear norm is the sum of its singular values, as provided by the tensor’s singular value decomposition (SVD).

Let ${∥A∥}_{*}$ denotes the nuclear norm of matrix A. Theorem in [42] states that the nuclear norm ${∥A∥}_{*}$ is the convex envelop of rank(A) within the unit ball (${∥A∥}_{F}\leq 1$). In our method, ∀d∈{s,t}, the prediction matrix $Y^{d}$ satisfies the following conditions:

(6) $$\begin{array}{cc} \; & \sum_{c=1}^{C}Y_{i,c}^{d}=1,\forall i\in \left\{1...N\right\};\;Y_{i,c}^{d}\geq 0,\forall i\in \left\{1...N\right\},c\in \left\{1...C\right\}. \end{array}$$

The Frobenius-norm of prediction matrix is calculated as:

(7) $$\begin{array}{c} ∥Y^{d}{∥}_{F}=\sqrt{\sum_{i=1}^{N}\sum_{c=1}^{C}{∥Y_{i,c}^{d}∥}^{2}}\leq \sqrt{\sum_{i=1}^{N}\left(\sum_{c=1}^{C}Y_{i,c}^{d}\right)\cdot \left(\sum_{c=1}^{C}Y_{i,c}^{d}\right)}\leq \sqrt{N}. \end{array}$$

Thus, in our situation, $∥Y^{d}{∥}_{F}\leq \sqrt{N}$, the theorem in [42] can be reused by scaling: the convex envelope of $rank(Y^{d})$ will be $∥Y^{d}{∥}_{*}/\sqrt{N}$, which is also proportional to $∥Y^{d}{∥}_{*}$. As the nuclear norm can be optimized efficiently, it is often adopted as the best convex approximation of the rank function in many literature [42,43] on rank optimization.

For Y, the maximum value of rank(Y) is r=min(2N,C). In [42,43,44], the relationship between ${∥Y∥}_{*}$ and Frobenius-norm ${∥Y∥}_{F}$ is as follows:

(8) $$\frac{1}{\sqrt{r}}{∥Y∥}_{*}\leq {∥Y∥}_{F}\leq {∥Y∥}_{*}\leq \sqrt{r}{∥Y∥}_{F}.$$

It shows that ${∥Y∥}_{*}$ and ${∥Y∥}_{F}$ could bound each other. In our method, we have

(9) $$\begin{array}{c} {∥Y∥}_{F}=\sqrt{\sum_{i=1}^{2N}\sum_{c=1}^{C}{∥Y_{i,c}∥}^{2}}\leq \sqrt{\sum_{i=1}^{2N}\left(\sum_{c=1}^{C}Y_{i,c}\right)\cdot \left(\sum_{c=1}^{C}Y_{i,c}\right)}\leq \sqrt{2N}. \end{array}$$

Then, the nuclear norm is upper bound by ${∥Y∥}_{*}\leq \sqrt{r}{∥Y∥}_{F}\leq \sqrt{2Nr}$. As a result, maximizing ${∥Y∥}_{*}$ will maximize ${∥Y∥}_{F}$, which represents the predicted diversity. Meanwhile, when ${∥Y∥}_{F}$ is maximized, the upper bound in (9) is achieved. It means that $\sum_{j}Y_{i,j}^{2}=\left(\sum_{j}Y_{i,j}\right)\cdot \left(\sum_{j}Y_{i,j}\right)$; then, we have $Y_{i,j_{1}}\cdot Y_{i,j_{2}}=0$ for $j_{1}\neq j_{2}$. Thus, each prediction $Y_{i}$ is a one-hot vector when ${∥Y∥}_{*}$ reaches the maximum, which indicates the predicted discriminability is also maximized. Replacing the rank(·) by nuclear norm, Equation (5) can be reformulated as:

(10) $$L_{dse}=\sum_{c=1}^{C}∥Y_{c}^{s}{∥}_{*}-{∥Y∥}_{*},$$

Provided that the class *c* exists in this mini-batch, $rank(Y_{c}^{s})\geq 1$. Thus, to avoid the prediction feature collapse being zero, we add the bound Δr on the intra-class rank, and we fix Δr=1. Thus, we re-write Equation (10) as

(11) $$L_{dse}=\sum_{c=1}^{C}\max(\Delta r,∥Y_{c}^{s}{∥}_{*}{)-∥Y∥}_{*},$$

### 3.3. Improving Transferability with Class-Conditional Distribution Alignment

DANN [16] applies Equation (3) to reduce the marginal distribution difference across-domain in feature space. In real scenarios, data distributions usually embody complex multi-modal structures due to the nature of multi-class classification. The multi-modal structure indicates that the dataset has multiple intrinsic attributes, e.g., contains images from different classes. Correspondingly, if the intrinsic attributes of the data pile up into a “mound”, it is called uni-modal. Aligning only the marginal feature distribution may fail to capture the multi-modal structures. Because even if the discriminator is completely confused, we cannot theoretically guarantee that the two different distributions are identical [45]. To address this issue, we apply the discriminative information conveyed from the task classifier prediction to align the class-conditional distribution in both feature and label spaces.

For the class-conditional distribution alignment in the feature space, we follow CDAN [17], which applies the discriminative information conveyed from the classifier prediction for conditional adversarial learning. It conditions the domain discriminator *D* on the classifier prediction with a multilinear map as follows:

(12) $$h^{s}=f^{s}⊗y^{s};\;h^{t}=f^{t}⊗y^{t},$$

where ⊗ is an operator of tensor product and $h^{s}$ ($h^{t}$) will be the new input of the conditional domain discriminator *D*. By taking advantage of the multilinear map, the updated adversarial learning loss can be written as:

(13) $$L_{adv}^{trans}=-\frac{1}{n_{s}}\sum_{i=1}^{n_{s}}\log\left(D\left(h_{i}^{s}\right)\right)-\frac{1}{n_{t}}\sum_{j=1}^{n_{t}}\log\left(1-D\left(h_{i}^{t}\right)\right).$$

The above loss can only align the class-conditional distribution ($P(X|Y_{c})$) across the domains in the feature space. We propose aligning the class-conditional label distribution $P(Y_{c})$ across two domains to enhance the transferability further. It is non-trivial to match $P(Y_{c}^{s})$ and $P(Y_{c}^{t})$ directly, as the target domain label is unavailable during training. We select the pseudo-labeled target samples with classification confidence higher than a certain threshold (0.95) to align the class-conditional label distribution. We exploit the constraint on matrix rank to force the classifier prediction of the same class in different domains to be embedded into the same subspace, that is, forcing the rank of each subspace of the source domain data to be approximated with the rank of the corresponding subspace of the target domain data. The class-conditional label distribution loss can be expressed as:

(14) $$L_{ld}=\sum_{c=1}^{C}\left(rank\left(Y_{c}^{s}\right)-rank\left({\hat{Y}}_{c}^{t}\right)\right),$$

where $Y_{c}^{s}$ is the sub-matrix of the source domain prediction belonging to class *c* and ${\hat{Y}}_{c}^{t}$ is the sub-matrix of the target prediction with a pseudo-label belonging to class *c*. We also apply the nuclear norm to achieve convex approximation of the rank, and Equation (14) can be reformulated as:

(15) $$L_{ld}=\sum_{c=1}^{C}(∥Y_{c}^{s}{∥}_{*}-∥{\hat{Y}}_{c}^{t}{∥}_{*}).$$

The underlying principle of this loss is to force the classifier prediction of the same class in different domains to be embedded into the same subspace, which can reduce the variation within each subspace. In this manner, the source and target domains of the same class will have consistent predictions, leading to better data alignment and transferability.

### 3.4. Overall Method and Optimization

Integrating all objectives together, the final objective can be outlined as follows:

(16) $$\begin{array}{cc} \; & \min_{F,G}L_{cls}+L_{adv}^{trans}+\beta L_{dse}+\lambda L_{ld}\\ \; & \max_{D}L_{adv}^{trans}, \end{array}$$

where β and λ are two trade-off hyper-parameters, $L_{cls}$ is the source classification loss, $L_{trans}^{adv}$ is the class-conditional feature distribution loss, $L_{dse}$ is the discriminative subspace embedding loss, and $L_{ld}$ is the class-conditional label distribution loss.

## 4. Experiments and Results

### 4.1. Datasets

**Office-31** [18]. It consists of three real-world image domains with 31 shared categories: Amazon (A), images are downloaded from Amazon online merchants; Webcam (W), images are obtained from low-resolution webcams; DSLR (D), images are obtained from a digital SLR camera with high-resolution. The total number of Office31 is 4652. Randomly select two domains as the source domain and the target domain, resulting in six cross-domain subtasks (A→W, ..., D →A, W →A).

**Office-Home** [46]. It consists of four significantly different data domains. These domains share 65 different categories from office and home scenes with a total number of 15,500. The four domains are: artistic images (denoted by Ar), which is an artistic depiction, such as sketches, paintings, and decorations of objects; clip art images (denoted by Cl), which constitute the image collection of clipart; product images (denoted by Pr), all its images have no background, similar to Amazon’s product images; real-world images (denoted by Rw) (all images are taken with a regular camera). This dataset has 12 adaptation sub-tasks; that is, Ar→Cl, ..., Rw→Pr.

**ImageCLEF-DA** (http://imageclef.org/2014/adaptation accessed on 1 November 2021) is a relatively small data set, which is the benchmark data set for ImageCLEF 2014 domain adaptation challenge. ImageCLEF-DA consists of three data domains, each of which shares 12 categories, and each category has 50 images. The three domains are from Caltech-256 (denoted by C), ImageNet ILSVRC 2012 (denoted by I), and Pascal VOC 2012 (denoted by P). Although the amount of data in each domain is very balanced, due to the small size of the domain, it is a relatively difficult dataset. There are six DA sub-tasks, that is, I→P, ..., P→C.

**VisDA2017** [47] is a very challenging dataset first proposed in the 2017 Visual Domain Adaptation Challenge, which contains two very distinct domains: synthetic images—images are rendered from 3D models with different angles and lighting; and real images, which are composed of natural images. It has a total of more than 280 K images with 12 shared classes in training, validation, and test set. The 12 shared classes are plane, bicycle (shortened to bcyle), bus, car, horse, knife, motorcycle (shortened to mcyle), person, plant, and skateboards (shortened to sktbrd). We treat the synthetic image dataset and the real image dataset as the source and target domains, respectively.

### 4.2. Baselines and Experimental Setup

To demonstrate the benefits of our MRE, we employ it on the two most popular adversarial adaptation networks: DANN [16] and CDAN [17]. We compared MRE with other adversarial DA networks and several SOTA deep DA methods: ADDA [20], which imposes an un-tied weight on the feature extractor and treated DANN as one of its special cases; JAN [10], which aligns the joint distribution; MCD [30], which does not explicitly use the discriminator, but apply two classifiers to implement adversarial training; MADA [35], which applies multiple domain discriminator to align the class-conditional distribution; MDD [48], which proposes a new and very effective distribution discrepancy measurement; BSP [14], which tries to preserve discriminability by penalizing the largest singular value of feature; BNM [39], which utilizes the F-norm and rank to improve feature discriminability and diversity; ALDA [31], which is a adversarial-based DA method; GVB [49], which applies the bridge to the generator and discriminator to progressively reduce the discrepancy across domains; *f*-DAL [27], which connects domain-adversarial learning with DA theory from the perspective of *f*-divergence minimization; CGDM [28], which, instead of directly manipulating the source and target domain features, minimizes their gradient difference; DWL [36], which dynamically balances the weight between feature alignment and feature discriminability in adversarial learning; MetaAlign [50], which regards distribution alignment and classification as the meta-train and meta-test tasks in a meta-learning scheme; and JUMBOT [51], which combines mini-batch strategy with unbalanced optimal transport to yield robust performance.

The code was implemented with PyTorch. For Office31, Office-Home, and ImageCLEF datasets, ResNet50 [15] pre-trained on ImageNet [52] was used as the backbone. For dataset VisDA2017, the backbone network will be replaced by the ResNet101 [15]. The network was trained by mini-batch stochastic gradient descent (SGD), and the momentum was set to 0.9. The learning rate schedule was the same as DANN [16] and CDAN [17]. Because both the domain discriminator and the classifier need to be trained from scratch, the learning rate was set to 10 times that of the backbone network. For data augmentation, some common operations, such as random flipping and random cropping, were employed. For Office31, Office-Home, and ImageCLEF datasets, the initial learning rate was 0.001. For the VisDA2017 dataset, the initial learning rate was 0.01. The batch size *N* was 36 for all datasets. We maintained the hyper-parameters β=0.1 and λ=0.01 as fixed. Our results are the average classification accuracy of three random experiments.

### 4.3. Results and Discussion

The results of Office-31 are displayed in Table 1. Our MRE significantly outperforms all comparison methods on most DA sub-tasks and achieves the best average result. Compared with the two baselines (DANN [16], CDAN [17]), MRE achieved a significant performance improvement on all subtasks, especially on difficult sub-tasks, D→A and W→A, in which there were significantly fewer source samples than the target domain. MRE achieved a final average accuracy improvement of 4.8% and 2.1% for DANN and CDAN, respectively, which demonstrates that domain adaptation can benefit from integrating matrix rank embedding into adversarial training to enhance the discriminability and transferability. Compared with the current SOTA DA methods, MRE still achieved competitive results.

Table 2 is the results on the ImageCLEF-DA dataset. The performance of MRE on the two baselines is improved. In Table 2, except for I→P and C→P, the accuracy of other sub-tasks are all over 90%, which shows that the sub-tasks are more challenging when P is the target domain. Nevertheless, our MRE achieved a significant improvement over the baseline in these two tasks. Compared with other methods, our MRE constitutes a relatively minor improvement since the images in ImageCLEF-DA are more visually similar, but the amount of data is very limited (600 for each domain), which may not be sufficient for training. Thus, the accuracies exhibited less room for improvement in all methods.

Table 3 shows the results of the Office-Home dataset. Compared with the two baselines, MRE achieved a significant performance improvement on all subtasks and achieved an average accuracy improvement of 9.0% and 5.9% for DANN [16] and CDAN [17], respectively. Compared with methods (BSP [14], ALDA [31], and BNM [39]) that focus on improving feature discriminability, our method has a significant improvement in terms of average accuracy. Compared with the current SOTA methods (GVB-GD [49], JUMBO [51]), our MRE with the CDAN significantly outperformed the comparison methods on eight sub-tasks and got the best average result. Especially, MRE is superior to MetaAlign on both baseline methods. It is noted that our MRE shows significant improvements compared with other DA methods when the artistic images (Ar) serve as the target domain. Since images in Ar within the same class have large differences, sub-tasks with Ar as the target domain are more challenging. Our MRE method still yielded larger improvement on such difficult DA sub-tasks, which highlights the power of our MRE.

Results of VisDA-2017 are displayed in Table 4. Compared with the two baselines DANN [16] and CDAN [17], MRE outperforms DANN (CDAN) in 9 (12) of 12 sub-tasks, and the average accuracy is improved by 14.2% and 8.5%, respectively. MRE provided the best performance in the final mean accuracy, surpassing the second-best (ALDA [31]) by 4.4%. Notably, ALDA learns the discriminative target features by generating a confusion matrix and trains the model in a self-training manner, while our MRE enhances transferability and discriminability simultaneously. Furthermore, according to the accuracy of each category, a substantial improvement was generated in the truck category. Compared to the other methods, which only focus on improving transferability or discriminability, our method achieved the best results, demonstrating that improving transferability and discriminability are equally important in DA.

### 4.4. Effectiveness Verification Experiments

**Ablation study**: To verify the effectiveness of each component in the objective function of MRE, ablation study was performed on the Office-Home dataset; the results are presented in Table 5. Our ablation study started with the very baseline method of DANN [16], which only aligns the marginal distribution without category information. Thereafter, we conducted a comparison with CDAN [17], which only aligns the class-conditional distribution of the data in the feature space. Subsequently, to investigate how the class-conditional distribution alignment in the label space aids in learning more transferable features, we removed the $L_{ld}$ loss in Equation (9) from main minimax problem in Equation (11), which was denoted as “MRE (w/o ld)”. To determine the effects of the proposed discriminative loss $L_{dse}$ in Equation (6), we removed Equation (6) from Equation (11), which was denoted as “MRE (w/o dse)”.

Table 5 demonstrates that CDAN provided a significant improvement over DANN, indicating that the discriminated multimodal structure information is very important in DA. MRE (w/o ld) outperformed CDAN, indicating the efficacy of our proposed discriminative adversarial learning. MRE (w/o dse) also outperformed CDAN, thereby demonstrating the effectiveness of aligning features and class conditional distribution of labels. MRE significantly outperformed MRE (w/o dse) and MRE (w/o ld), confirming the efficacy of the proposed simultaneous improvement in the discriminability and transferability.

**Figure 3:**
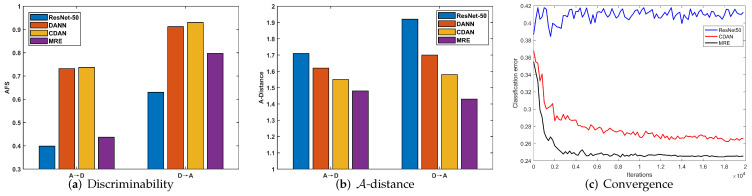
Discriminability and transferability of different methods for tasks A→D and D→A: (**a**) discriminability and (**b**) A-distance. (**c**) Convergence on adaptation task W→A.

**Discriminability**: We investigated the discriminability of different methods by calculating the AFS [19]. As mentioned previously, the AFS serves as an effective indicator of discriminability. A lower Fisher value indicates that the features are more discriminative. The results of sub-tasks A→D and D→A are presented in Figure 3a. Comparing ResNet-50 with DANN and CDAN, although adversarial domain adaptation methods can enhance the transferability, as they achieve better performance in Table 1, the discriminability of DANN and CDAN is reduced, while our MRE can not only significantly enhance the discriminability but also preserve transferability.

**Distribution discrepancy**: In DA, the cross-domain distribution discrepancy is commonly measured by A-distance [3], which is calculated as $d_{A}=2\left(1-2\epsilon \right)$. We denote ϵ as the test error of a classifier, which is trained to discriminate whether a feature vector *v* comes from the source domain or the target domain, where *v* is the feature extracted from a learned DA feature extractor. We compared our proposed MRE with ResNet-50 [15], DANN [16], and CDAN [17] on the subtasks A→D and D→A in the Office31 dataset. As shown in Figure 3b, the A-distances of DANN, MRE, and CDAN were smaller than that of ResNet-50, indicating that adversarial DA enables significantly reduce cross-domain distribution discrepancy. The A-distance of MRE is the smallest among DANN, CDAN, and MRE, indicating that the features extracted by our MRE show better transferability.

**Convergence**: To verify the convergence of ResNet-50 [15], CDAN [17], and our MRE, we conducted an experiment on the sub-task W→A in the Office31 dataset. Figure 3c presents the result. The test error in Figure 3c is equal to (1.0—accuracy). The value of ResNet-50 is the target domain test error by the network trained only with the source domain data. Because target domain data does not present in the training of ResNet50, the learned parameter is irrelevant to the target domain. As a result, its test error in the target domain fluctuates in a small range. Our MRE yielded faster convergence than CDAN.

**Figure 4:**
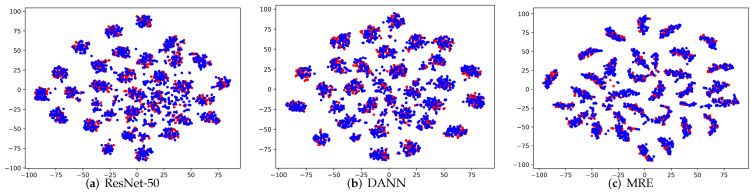
(**a**–**c**) Visualization on task A→D (best viewed in color), where red indicates source samples and blue denotes target samples.

**Visualization**: To verify the clustering and separation characteristic of the extracted features, we apply the commonly used t-SNE [53] to visualize the activations from different feature extractors. We conducted an experiment on the subtask A→D and compared our MRE method with ResNet-50 and DANN. As can be seen from the results in Figure 4a–c, for the ResNet-50, there is a considerable proportion of the features are not aligned, the intra-class distance is relatively large, and the inter-class distance is relatively small. Comparing DANN with ResNet-50, the source domain and target features of DANN are better aligned, but its intra-class distance is still large. In MRE, the features were well aligned and exhibited better intra-class clustering and inter-class separation. This demonstrates the effectiveness of our MRE in aligning the class-conditional distributions in both feature and label space, and in learning a more discriminated target model.

**Hyper-parameter analysis**: There are three hyper-parameters—β, λ, and the threshold th—where th is used to select target samples with higher confidence. β and λ are two trade-off parameters, which are used to control the discriminative subspace embedding loss and the class-conditional label distribution loss, respectively. A case study on dataset Office-31 was conducted to investigate the sensitivity of th, β, and λ. For each parameter, a set of reasonable values was selected to form a discrete candidate set, for th∈ {0.85, 0.90, 0.95, 0.97, 0.99}, for β∈{0.01, 0.05, 0.1, 0,2, 0.5}, and for λ∈ {0.001, 0.005, 0.01, 0.05, 0.1}. The results are presented in Table 6. When the value of th is greater than 0.9, th is insensitive. We fix th=0.95. For β and λ, our MRE achieves the best result with β=0.1 and λ=0.01. From the results, as long as the parameters are within the feasible range, our MRE is robust to different settings. One can tune the hyper-parameter by IWCA [54] for different applications.

**Runtime comparison**: We conduct experiments on sub-task of A→W in Office-31 dataset to compare the runtime. All experiments were run on the same machine (Linux version 4.15.0-20-generic, Ubuntu 7.3.0-16ubuntu3, python version = 1.3.1, CUDA version = 10.0.130, GPU = Tesla V100-PCIE-32GB). The batch size of all experiments is set to 36. CDAN is our baseline network. Table 7 reports the total runtime required for each algorithm to train 20,000 iterations. In Table 7, “MRE(w/o $L_{ld}$)” means MRE without the $L_{ld}$ loss and “MRE(w/o $L_{dse}$)” means MRE without the discriminative loss $L_{dse}$. Compared to the baseline CDAN, our method has only a slight increase in computational cost. Our objective function contains four matrix nuclear-norm operators, which are calculated as the sum of matrix singular-values. Singular value decomposition (SVD) is very time-consuming in traditional machine learning. However, our calculation of SVD is based on mini-batches. Meanwhile, we calculate the SVD in label space, which has much lower dimensions compared to the feature space. Therefore, our method is computationally effective.

## 5. Conclusions and Discussion

In this paper, we conduct an experiment to confirm that the discriminability of target domain features is inevitably suppressed during the process of adversarial learning. Further, we propose an approach for adversarial DA with matrix rank embedding as the key learning criterion to simultaneously enhance discriminability and transferability. We force data of the same class to have a low-rank structure and data of different classes to have a high-rank structure, thereby resulting in improved discriminability. We also force data from the same class but different domains to have an approximate low-rank structure, aligning the class-conditional distribution in label space, resulting in enhanced transferability. Our method is general and can be combined with most classification algorithms since the proposed loss function is directly applied to the softmax probability matrix in classification. Thus, it can be considered a plug-in module in the classification networks. Second, our method has more advantages in challenging tasks. Experiments show that our method performs similar to CDAN in simple datasets and outperforms CDAN significantly in challenging datasets like Office-Home.

Nevertheless, our method also has some limitations. First, we use a nuclear-norm-based objective for optimization, thus bringing computation burden in calculating SVD. Second, we introduce three additional hyper-parameters that need to be tuned in experiments. In our paper, we use the grid search to set our hyper-parameter. In practice, some commonly used machine learning hyper-parameter optimization methods, such as random search and Bayesian model-based optimization, can also be used to search hyper-parameters. Third, the target pseudo-labels are not always correct. These misclassified pseudo-labels in the target domain may affect the class-conditional label distribution alignment. Such a problem can be improved by designing better pseudo-labeling mechanisms in future work. Future research may focus on addressing the above drawbacks and limitations.

## Figures and Tables

**Table 1:** Classification results (accuracy %) on Office-31 dataset with ResNet-50 as the backbone. ↑ denotes an increase in performance. The bold number indicates the best performance.

Methods	A→W	D→W	W→D	A→D	D→A	W→A	Avg
ResNet50	68.4 ± 0.2	96.7 ± 0.1	99.3 ± 0.1	68.9 ± 0.2	62.5 ± 0.3	60.7 ± 0.3	76.1
ADDA	86.2 ± 0.5	96.2 ± 0.3	98.4 ± 0.3	77.8 ± 0.3	69.5 ± 0.4	68.9 ± 0.5	82.9
MADA	90.1 ± 0.1	97.4 ± 0.1	99.6 ± 0.1	87.8 ± 0.2	70.3 ± 0.3	66.4 ± 0.3	85.2
MDD	94.5 ± 0.3	98.4 ± 0.1	**100.0 ± 0.0**	93.5 ± 0.2	74.6 ± 0.3	72.2 ± 0.1	88.9
BSP	93.3 ± 0.2	98.2 ± 0.2	**100.0 ± 0.0**	93.0 ± 0.2	73.6 ± 0.3	72.6 ± 0.3	88.5
BNM	92.8 ± 0.1	98.8 ± 0.1	**100.0 ± 0.0**	92.9 ± 0.2	73.5 ± 0.2	73.8 ± 0.3	88.6
ALDA	**95.6 ± 0.5**	97.7 ± 0.1	**100 ± 0.0**	94.0 ± 0.4	72.2 ± 0.4	72.5 ± 0.2	88.9
GVB-GD	94.8 ± 0.5	98.7 ± 0.3	**100.0 ± 0.0**	95.0 ± 0.4	73.4 ± 0.3	73.7 ± 0.4	89.3
*f*-DAL	95.4 ± 0.7	98.8 ± 0.1	**100.0 ± 0.0**	93.8 ± 0.4	74.9 ± 1.5	74.2 ± 0.5	89.5
GVB+MetaAlign	93.0 ± 0.5	98.6 ± 0.0	**100.0 ± 0.0**	94.5 ± 0.3	75.0 ± 0.3	73.6 ± 0.0	89.2
DWL	89.2	**99.2**	**100.0**	91.2	73.1	69.8	87.1
DANN	82.0 ± 0.4	96.9 ± 0.2	99.1 ± 0.1	79.7 ± 0.4	68.2 ± 0.4	67.4 ± 0.5	82.2
DANN+MRE	91.9 ± 0.6 ↑	98.6 ± 0.0 ↑	99.3 ± 0.1 ↑	88.6 ± 0.2 ↑	71.7 ± 0.3 ↑	72.1 ± 0.3 ↑	87.0 ↑
CDAN	93.1 ± 0.1	98.6 ± 0.1	**100.0 ± 0.0**	92.9 ± 0.2	71.0 ± 0.3	70.3 ± 0.3	87.7
CDAN+MRE	94.3 ± 0.4 ↑	98.6 ± 0.2	**100.0 ± 0.0**	**95.5 ± 0.2** ↑	**75.8 ± 0.4** ↑	**74.6 ± 0.4** ↑	**89.8** ↑

**Table 2:** Classification results (accuracy %) of different methods on ImageCLEF-DA dataset. The backbone network is ResNet-50. ↑ denotes an increase in performance. The bold number indicates the best performance.

Methods	I→P	P→I	I→C	C→I	C→P	P→C	Avg
ResNet50	74.8 ± 0.3	83.9 ± 0.1	91.5 ± 0.3	78.0 ± 0.2	65.5 ± 0.3	91.2 ± 0.3	80.7
DAN	74.5 ± 0.4	82.2 ± 0.2	92.8 ± 0.2	86.3 ± 0.4	69.2 ± 0.4	89.8 ± 0.4	82.5
JAN	76.8 ± 0.4	88.0 ± 0.2	94.7 ± 0.2	89.5 ± 0.3	74.2 ± 0.3	91.7 ± 0.3	85.8
ADDA	75.5	88.2	96.5	89.1	75.1	92.0	86.0
MCD	77.3	89.2	92.7	88.2	71.0	92.3	85.1
MADA	75.0 ± 0.3	87.9 ± 0.2	96.0 ± 0.3	88.8 ± 0.3	75.2 ± 0.2	92.2 ± 0.3	85.9
BNM	78.5 ± 0.4	91.5 ± 0.2	95.8 ± 0.2	91.8 ± 0.2	76.8 ± 0.2	95.0 ± 0.3	88.2
CGDM	78.7 ± 0.2	**93.3 ± 0.1**	97.5 ± 0.3	**92.7 ± 0.2**	**79.2 ± 0.1**	**95.7 ± 0.2**	89.5
DANN	75.0 ± 0.3	86.0 ± 0.3	96.2 ± 0.4	87.0 ± 0.5	74.3 ± 0.5	91.5 ± 0.6	85.0
DANN+MRE	77.8 ± 0.4 ↑	92.7 ± 0.3 ↑	96.5 ± 0.2 ↑	92.7 ± 0.2 ↑	77.5 ± 0.2 ↑	94.2 ± 0.4 ↑	88.6 ↑
CDAN	77.7 ± 0.3	90.7 ± 0.2	97.7 ± 0.3	91.3 ± 0.3	74.2 ± 0.2	94.3 ± 0.3	87.7
CDAN+MRE	**79.7 ± 0.4** ↑	92.9 ± 0.2 ↑	**97.9 ± 0.3** ↑	**92.7 ± 0.4** ↑	**79.2 ± 0.2** ↑	95.0 ± 0.5 ↑	**89.8** ↑

**Table 3:** Classification results (accuracy %) of different methods on Office-Home dataset. The backbone network is ResNet-50. ↑ denotes an increase in performance. The bold number indicates the best performance.

Methods	Ar→Cl	Ar→Pr	Ar→Rw	Cl→Ar	Cl→Pr	Cl→Rw	Pr→Ar	Pr→Cl	Pr→Rw	Rw→Ar	Rw→Cl	Rw→Pr	Avg
ResNet50	34.9	50.0	58.0	37.4	41.9	46.2	38.5	31.2	60.4	53.9	41.2	59.9	46.1
DAN	43.6	57.0	67.9	45.8	56.5	60.4	44.0	43.6	67.7	63.1	51.5	74.3	56.3
JAN	45.9	61.2	68.9	50.4	59.7	61.0	45.8	43.4	70.3	63.9	52.4	76.8	58.3
MDD	54.9	73.7	77.8	60.0	71.4	71.8	61.2	53.6	78.1	72.5	60.2	82.3	68.1
BSP	52.0	68.6	76.1	58.0	70.3	70.2	58.6	50.2	77.6	72.2	59.3	81.9	66.3
ALDA	53.7	70.1	76.4	60.2	72.6	71.5	56.8	51.9	77.1	70.2	56.3	82.1	66.6
BNM	56.2	73.7	79.0	63.1	73.6	74.0	62.4	54.8	80.7	72.4	58.9	83.5	69.4
MDD+Implicit	56.2	**77.9**	79.2	64.4	73.1	74.4	64.2	54.2	79.9	71.2	58.1	83.1	69.5
GVB-GD	57.0	74.7	79.8	64.6	74.1	74.6	65.2	55.1	**81.0**	74.6	59.7	**84.3**	70.4
f-DAL	54.7	71.7	77.8	61.0	72.6	72.2	60.8	53.4	80.0	73.3	60.6	83.8	68.5
JUMBOT	55.2	75.5	**80.8**	65.5	74.4	74.9	65.2	52.7	79.2	73.0	59.9	83.4	70.0
DANN+MetaAlign	48.6	69.5	76.0	58.1	65.7	68.3	54.9	44.4	75.3	68.5	50.8	80.1	63.3
CDAN+MetaAlign	55.2	70.5	77.6	61.5	70.0	70.0	58.7	55.7	78.5	73.3	**61.0**	81.7	67.8
DANN	45.6	59.3	70.1	47.0	58.5	60.9	46.1	43.7	68.5	63.2	51.8	76.8	57.6
DANN+MRE	52.8 ↑	69.7 ↑	76.3 ↑	58.7 ↑	70.9 ↑	70.8 ↑	58.6 ↑	51.8 ↑	78.6 ↑	70.6 ↑	57.3 ↑	82.7 ↑	66.6 ↑
CDAN	50.8	68.3	74.9	58.4	70.6	70.1	54.8	48.7	76.6	70.3	57.7	81.6	65.2
CDAN+MRE	**57.8** ↑	75.2 ↑	79.5 ↑	**65.9** ↑	**74.8** ↑	**75.0** ↑	**66.8** ↑	**56.6** ↑	80.8 ↑	**75.8** ↑	60.2 ↑	**84.3** ↑	**71.1** ↑

**Table 4:** Classification results (accuracy %) of different methods on VisDA2017 dataset. ResNet-101 is the backbone network. ↑ denotes an increase in performance. The bold number indicates the best performance.

Methods	Plane	Bcybl	Bus	Car	Horse	Knife	Mcyle	Person	Plant	Sktbrd	Train	Truck	Avg
ResNet101	55.1	53.3	61.9	59.1	80.6	17.9	79.7	31.2	81.0	26.5	73.5	8.5	52.4
DAN	87.1	63.0	76.5	42.0	90.3	42.9	85.9	53.1	49.7	36.3	85.8	20.7	61.1
MCD	87.0	60.9	83.7	64.0	88.9	79.6	84.7	76.9	88.6	40.3	83.0	25.8	71.9
BSP	92.4	61.0	81.0	57.5	89.0	80.6	90.1	77.0	84.2	77.9	82.1	38.4	75.9
ALDA	93.8	74.1	82.4	69.4	90.6	87.2	89.0	67.6	**93.4**	76.1	87.7	22.2	77.8
DWL	90.7	80.2	86.1	67.6	**92.4**	81.5	86.8	78.0	90.6	57.1	85.6	28.7	77.1
DANN	81.9	**77.7**	82.8	44.3	81.2	29.5	65.1	28.6	51.9	54.6	82.8	7.8	57.4
DANN+MRE	90.0 ↑	69.5	75.9	48.2 ↑	86.8 ↑	28.8	91.5 ↑	75.9 ↑	91.1 ↑	66.9 ↑	88.0 ↑	**46.3** ↑	71.6 ↑
CDAN	85.2	66.9	83.0	50.8	84.2	74.9	88.1	74.5	83.4	76.0	81.9	38.0	73.7
CDAN+MRE	**95.1** ↑	71.7 ↑	**85.6** ↑	**71.2** ↑	91.4 ↑	**89.5** ↑	**92.9** ↑	**80.0** ↑	91.2 ↑	**83.3** ↑	**88.1** ↑	**46.3** ↑	**82.2** ↑

**Table 5:** Ablation study on Office-Home dataset. ResNet-50 is the backbone network. The bold number indicates the best performance.

Methods	Ar→Cl	Ar→Pr	Ar→Rw	Cl→Ar	Cl→Pr	Cl→Rw	Pr→Ar	Pr→Cl	Pr→Rw	Rw→Ar	Rw→Cl	Rw→Pr	Avg
DANN	45.6	59.3	70.1	47.0	58.5	60.9	46.1	43.7	68.5	63.2	51.8	76.8	57.6
CDAN	50.8	68.3	74.9	58.4	70.6	70.1	54.8	48.7	76.6	70.3	57.7	81.6	65.2
CDAN+MRE (w/o dse)	49.4	71.2	77.9	63.1	70.8	73.0	60.8	48.7	79.3	72.5	54.7	82.3	67.0
CDAN+MRE (w/o ld)	56.7	74.8	79.2	65.3	74.2	74.7	64.9	56.3	**81.5**	73.4	59.2	83.7	70.3
CDAN+MRE	**57.8**	**75.2**	**79.5**	**65.9**	**74.8**	**75.0**	**66.8**	**56.6**	80.8	**75.8**	**60.2**	**84.3**	**71.1**

**Table 6:** Results (%) on Office-31 for sensitivity of th, α and λ.

th	0.85	0.90	0.95	0.97	0.99
Avg	87.7	89.8	89.8	89.8	89.0
β	0.01	0.05	0.1	0.2	0.5
Avg	88.7	89.2	89.8	88.9	87.7
λ	0.001	0.005	0.01	0.05	0.1
Avg	86.8	88.5	89.8	87.1	88.2

**Table 7:** Running time (s) comparison on task of A→W.

Methods	$L_{\mathrm{dse}}$	$L_{\mathrm{ld}}$	Runtime	Relative Runtime
CDAN	×	×	7257 s	100%
MRE(w/o $L_{ld}$)	√	×	7363 s	101.46%
MRE(w/o $L_{dse}$)	×	√	7391 s	101.85%
MRE	√	√	7452 s	102.69%

## Data Availability

The data presented in this study are openly and permanently available in OSF.IO at https://osf.io/ajsc8/ (accessed on 3 November 2021). All the datasets used in this study are public datasets and are permanently available at https://github.com/jindongwang/transferlearning/tree/master/data (accessed on 3 November 2021).
